# Cholangiocarcinoma cells secrete soluble factors that activate Jak/STAT signal transduction and promote MDSC expansion

**DOI:** 10.1186/2051-1426-2-S3-P271

**Published:** 2014-11-06

**Authors:** Jennifer Yang, Kaitlin Keenan, Thomas Mace, Matthew Ferren, Tanios Bekaii-Saab, James Fuchs, Eric Schwartz, Chenglong Li, Jiayuh Lin Pui-Kai Li, Gregory Lesinski

**Affiliations:** 1 The Ohio State University, Columbus, OH, USA

## 

Cholangiocarcinoma (CC) is a universally lethal disease that responds poorly to chemo- and immunotherapy. Patients with CC typically display elevated systemic interleukin-6 (IL-6) and systemic expansion of myeloid-derived suppressor cells (MDSCs). Interestingly, both of these processes involve activation of the STAT3 transcription factor. We hypothesize that tumor-derived cytokines may act via distinct signaling pathways, such as STAT3 to limit immune-mediated recognition of CC. In a panel of n = 7 human CC cell lines with diverse genetic profiles, constitutive phosphorylation of STAT3, STAT5, ERK and Akt was observed. Consistent with an important role for STAT3 signaling in this disease, treatment of CC cell lines with FLLL100, a novel, small-molecule, STAT3 inhibitor led to significant growth inhibition and apoptosis in a concentration dependent manner. Target inhibition of STAT3 and PARP processing were confirmed by immunoblot analysis of lysates from human CC cell lines. Supernatants from CC cells secreted various immunomodulatory cytokines including IL-6, GM-CSF, IL-8, and VEGF. Culture of CC supernatants with human peripheral blood mononuclear cells also promoted the expansion of CD33+CD11b+HLADRlow MDSC that could functionally suppress proliferation of autologous CD3+ T lymphocytes. To gain greater insight into the mechanisms by which human CC acted upon immune cells to promote MDSC expansion, we evaluated signal transduction events upon exposure of PBMCs to CC supernatants. These data indicated that supernatants from 7/7 CC cell lines induced Tyr705-phosphorylation of STAT3, while supernatants from 4/7 CC cell lines induced phosphorylation of STAT5. In the 4 cell lines tested to date, antibody-mediated neutralization of IL-6 from CC supernatants inhibited the ability to phosphorylate STAT3, but not STAT5. These data indicate that IL-6 was the predominant cytokine responsible for STAT3 activation. However, neutralization of IL-6 did not inhibit the ability of CC supernatants to promote expansion of MDSC *in vitro*. These data indicate that CC-derived soluble factors other than IL-6 may contribute to specific mechanisms of immunosuppression, such as MDSC expansion that are being considered as targets to enhance immunotherapy in this disease.

